# Engineering HER2-targeted biparatopic antibodies to promote receptor internalization and restore antitumor efficacy

**DOI:** 10.3389/fimmu.2025.1711433

**Published:** 2025-11-07

**Authors:** Xinlin Liu, Wanpeng Yu, Yihuan Wang, Dongming Xing, Haiming Huang, Wenjing Zhu, Peng Sun

**Affiliations:** 1 Department of Hepatobiliary and Pancreatic Surgery, The Affiliated Hospital of Qingdao University, Qingdao, China; 2 Qingdao Cancer Institute, Qingdao, China; 3 Qingdao Medical College, Qingdao University, Qingdao, China; 4 Noventi Biopharmaceuticals Co., Ltd, Shanghai, China; 5 Medical Research Department, Qingdao Hospital, University of Health and Rehabilitation Sciences (Qingdao Municipal Hospital), Qingdao, China

**Keywords:** biparatopic antibody, HER2, nanobody, trastuzumab-resistance, non-overlapping epitopes

## Abstract

HER2 is a well-established oncogenic driver in breast, gastric, and other solid tumors. While HER2-targeted therapies such as trastuzumab and pertuzumab have improved clinical outcomes, resistance, particularly to trastuzumab, remains a major therapeutic challenge. Here, we engineered two IgG-VHH biparatopic antibodies (bpAbs), A9B5-Bs-5 and A9B5-Bs-7, incorporating an ECD I-binding nanobody A9B5 with the IgG scaffolds. These bpAbs target non-overlapping epitopes on the HER2 extracellular domain, promoting rapid receptor internalization and demonstrating superior antitumor activity compared to the trastuzumab and pertuzumab combination in trastuzumab-resistant tumor cells. Structural modeling suggests that both bpAbs engage HER2 in a *trans*-binding mode, leading to receptor clustering and interference with ligand-driven HER2 heterodimerization. These findings demonstrate that epitope-guided biparatopic antibody design can enhance HER2 downregulation and restore sensitivity to HER2-targeted therapy *in vitro*, providing a strategy for the development of next-generation receptor-targeted biologics.

## 1 Introduction

Human epidermal growth factor receptor 2 (HER2), a member of the epidermal growth factor receptor (EGFR or HER) family, is overexpressed in various solid tumors and is associated with aggressive disease and poor prognosis (1–4). HER2-targeted therapies have markedly improved outcomes in patients with HER2-positive cancers. Among these, the monoclonal antibodies trastuzumab and pertuzumab, in combination with chemotherapy, form a standard-of-care regimen that significantly prolongs survival (5–7). Trastuzumab binds to the extracellular domain (ECD) IV of HER2, inhibiting ligand-independent HER2-HER3 dimerization and mediating antibody-dependent cellular cytotoxicity (ADCC), while pertuzumab disrupts ligand-driven HER2 heterodimerization by targeting the dimerization arm (DA) of ECD II (8, 9). Nevertheless, both intrinsic and acquired resistance, particularly to trastuzumab, remain significant clinical obstacles (10–15).

Resistance to anti-HER2 therapies arises through diverse mechanisms, including HER2 mutations, compensatory signaling, receptor masking, downregulation, and intertumoral heterogeneity (10, 13, 16–21). These complexities highlight the need for therapeutic strategies capable of simultaneously blocking multiple signaling axes and addressing tumor heterogeneity. Bispecific antibodies (bsAbs), which bind two different antigens or epitopes, offer a promising approach (22, 23). Through combinatorial mechanisms of action (MOAs), bsAbs can enhance receptor blockade, promote internalization, and counteract escape pathways that limit the efficacy of monospecific antibodies (24–26). Biparatopic antibodies (bpAbs), a subclass of bsAbs that recognize non-overlapping epitopes on the same antigen, have demonstrated unique functional advantages. For instance, the IgG-like bpAb zanidatamab, which targets HER2 ECD II and ECD IV, induces receptor clustering and complement-dependent cytotoxicity (CDC), and has shown encouraging efficacy in early-stage clinical studies (27–32). These findings suggest that dual-epitope engagement can elicit distinct and therapeutically beneficial biological responses compared to parental monoclonals. However, clinically advanced anti-HER2 bpAbs predominantly focus on ECD II and IV, which may limit their effectiveness against tumors harboring mutations in these regions (33, 34). For instance, ECD II mutations such as S310F/Y, associated with drug resistance and metastasis, can abolish pertuzumab binding (35).

The dynamic structural attributes of HER2, particularly its capacity for dimerization, underlies both its oncogenic activity and its resistance to targeted therapies (36, 37). While the DA of ECD II mediates dimerization, recent structural studies of HER2-EGFR and HER2-HER3 complexes have identified a critical role for the ECD I-III interface in forming the binding pocket for dimerization partners (38, 39). Targeting ECD I or III, in combination with trastuzumab or pertuzumab, has shown synergistic efficacy in overcoming therapeutic resistance (40–48). We previously identified a high-affinity ECD I-binding nanobody A9B5, which exhibits potent synergy with trastuzumab in resistant models (49). These findings highlight the potential of bpAbs designed to target novel alternative epitopes to comprehensively block HER2-driven signaling.

Antibody format is another key determinant of bsAb function, stability, and developability. To date, over 50 bsAb formats have been developed (50). Among them, IgG-VHH fusions incorporating single domain (VHH) nanobodies are attractive due to their high solubility, stability, and reduced risk of mispairing (51, 52). In this study, we engineered and evaluated a panel of IgG-VHH bpAbs incorporating the A9B5 nanobody. Two lead candidates, A9B5-Bs-5 and A9B5-Bs-7, demonstrated potent HER2 internalization and antitumor efficacy superior to the combination of trastuzumab and pertuzumab in trastuzumab-resistant models.

## 2 Methods

### 2.1 Cell lines, antibodies, and biological material

NCI-N87, MCF-7, JIMT-1, and BT474 were sourced from ATCC. Expi 293 cells were purchased from Thermo Fisher Scientific. Trastuzumab and pertuzumab were produced in-house. A non-specific IgG antibody, which was obtained from Beyotime (A7001), was used as a negative control.

### 2.2 Construction of bpAbs and chimeric proteins

The sequences encoding HER2-targeting nanobodies were cloned into either the N- or C-terminus of the heavy or light chains of trastuzumab or pertuzumab within pSCSTa expression vectors. Vectors encoding nanobody-fused heavy chains and corresponding light chains, or nanobody-fused light chains and corresponding heavy chains, were transiently transfected into Expi293 cells using standard protocols. After 168 h of culture, the supernatant was harvested and purified by Protein A affinity chromatography using AT Protein A Diamond Plus resin (BestChrom, AA402305). The eluted antibodies were buffer-exchanged into PBS. Antibody concentrations were determined by BCA assay, and purity was assessed via SDS-PAGE.

Chimeric HER2-ECD proteins (HER2-mD1, HER2-mD2, HER2-mD3, and HER2-mD4) were constructed as previously described (49). Briefly, the extracellular domains I (T23–R217), II (T218–C342), III (Y344–A510), and IV (C511–T652) of the HER2 protein (UniProt P04626) were replaced with their respective murine homologous domains (UniProt P70424). DNA sequences encoding these chimeric proteins were cloned into a pSCSTa vector containing a C-terminal Fc tag and transiently transfected into 293T cells. Protein expression and purification were performed as described for the above antibodies.

### 2.3 Growth inhibition assays

Tumor cells were seeded into 96-well plates at approximately 2 × 10^3^ cells per well. Plates were incubated overnight at 37°C with 5% CO_2_. The next day, antibodies were serially diluted using a 1:5 dilution series. Diluted antibodies were added to the wells and incubated with the cells for five days. Cell viability was assessed using the Cell Counting Kit-8 (CCK-8, Dojindo) according to the manufacturer’s protocol. After incubation, the supernatant was removed. Cells were then treated with fresh medium containing 10% (v/v) CCK-8 reagent. Plates were incubated at 37°C with 5% CO_2_ for 2 h, and then absorbance at 450 nm was recorded using a BioTek plate reader. For ligand-dependent assays, tumor cells were stimulated with either 1 nM HRG (SinoBiological, 11609-HNCH) or 5 nM EGF (SinoBiological, GMP-10605-HNAE). Cell viability was calculated as a percentage relative to untreated control cells. Background values were subtracted. Dose–response curves were fitted using the “log(inhibitor) vs. response—variable slope (four parameters)” model in GraphPad Prism. EC_50_ values were calculated based on curve fitting. Statistical significance was tested using one-way or 2-way ANOVA. Comparisons were made between antibody-treated groups at either 150 nM or 30 nM. The corresponding p-values were reported.

### 2.4 Size-exclusion chromatography (HPSEC)

Samples of anti-HER2 agents were loaded onto a TSK Gel G 3000 pwxl (7.8 × 300 mm; TOSOH, Tokyo, Japan) equilibrated with PBS. Proteins separation was performed using Agilent 1200 series system (Agilent Technologies, Santa Clara, CA). The flow rate was set to 0.5 mL per minute. Elution was monitored by UV absorbance at 280 nm.

### 2.5 Enzyme-linked immunosorbent assay

To identify the binding epitopes of HER2-targeting bpAbs, 96-well plates were coated with either wild-type or chimeric HER2-ECD proteins (100 ng per well) in PBS and incubated overnight at 4 °C. Plates were subsequently blocked with 2% NFDM at room temperature for 2 h. Threefold serial dilutions of bpAbs, anti-HER2 nanobodies (His-tag), or other anti-HER2 antibodies were added and incubated at room temperature for 1 h. For detection, HRP-conjugated His-Tag monoclonal antibody (Proteintech, HRP-66005) was used for nanobodies, and the HRP-conjugated mouse anti-human IgG Fc antibody (GenScript, A01854) was used for IgG-based antibodies. After a 30-minute incubation, the wells were washed, and TMB substrate was added for color reaction. The reaction was quenched with 1M H_3_PO_4_, and the absorbance at 450 nm (reference 620 nm) was measured using an automated ELISA reader (BioTek). Data was analyzed using GraphPad Prism 10.2.0. Curve fitting was performed by nonlinear regression using the “log(agonist) vs. response - variable slope (four parameters)” model to determine the EC_50_ value.

### 2.6 Internalization by fluorescence activated cell sorter

NCI-N87 or BT474 cells in exponential growth were harvested and resuspended using PBS + 2% FBS. Cell viability was assessed using an automated cell counter (Countstar BioTech). 3-5 × 10^4^ viable cells per well were seeded in 96-well plates. Cells were incubated with anti-HER2 antibodies on ice for 1 h, followed by two washes with PBS to remove unbound antibodies. An aliquot of cells was maintained on ice, while the rest were incubated at 37°C for 0.5 or 4 h. Following incubation, cells were washed and fixed with 4% paraformaldehyde (PFA) for 20 min at room temperature. Cells were then stained with PE-conjugated anti-human IgG Fc antibody (Abcam, 98596; 1:1000 dilution) at 4°C for 30 min. The Beckman Coulter flow cytometer was used to measure median fluorescence intensity (MFI) in the PE-channel. The antibody-receptor complexes internalization was calculated as the percentage loss of MFI at 37°C relative to the signal measured on ice. Data was analyzed using GraphPad Prism v10.2.0.

### 2.7 Internalization by confocal imaging

NCI-N87 or MCF-7 cells in exponential growth were seeded onto coverslips and cultured overnight at 37°C/5% CO_2_. Cells were treated with 50 nM antibodies at 4°C for 1 h. Unbound antibodies were removed by washing with PBS supplemented with 2% FBS, followed by incubation at 37°C for 4 h. After washing, cells were fixed with 4% PFA and permeabilized with Triton X-100. Blocking was performed in PBS containing 10% goat serum (Solarbio, SL038) at room temperature for 1 h. Cells were stained with DyLight^®^ 488-conjugated goat anti-human IgG Fc antibody (green; Abcam, ab98619) to visualize antibody-receptor complexes, and with anti-LAMP1 antibody (Abcam, ab25630), followed by Alexa-Fluor 647-labeled goat anti-mouse IgG H&L (magenta; Abcam, ab150115) to label lysosomes. The 4′,6-diamidino-2-phenylindole (DAPI, blue; Beyotime, C1002) was used for nuclear staining. Coverslips were treated with an antifade mounting medium (Beyotime, P0126). Confocal imaging was performed using a Nikon A1 confocal microscope and analyzed with NIS-Elements Viewer software. Fluorescence intensity from three channels was quantified using ImageJ. Mean intensity values were analyzed using GraphPad Prism v10.2.0.

### 2.8 HER2 binding by fluorescence activated cell sorter

FACS was performed on HER2-expressing tumor cell lines with differential responses to trastuzumab: NCI-N87 (trastuzumab-sensitive), MCF-7 (trastuzumab-resistant), and JIMT-1 (trastuzumab-resistant). Tumor cells in the exponential growth were harvested and resuspended in PBS supplemented with 2% FBS. A total of 1 × 10^5^ cells per well were incubated with diluted anti-HER2 antibodies for 1 h on ice. Then, cells were washed and stained with PE-conjugated anti-human IgG Fc secondary antibody (Abcam, 98596; 1:1000). MFI in the PE-channel was measured using a Beckman Coulter flow cytometer. Binding data was analyzed using GraphPad Prism 10.2.0.

### 2.9 Structure modeling

The structure of A9B5-HER2 complex was predicted using Alphafold 3 (https://alphafoldserver.com/) following the server’s guidelines (53). Briefly, the amino acid sequences of the A9B5 and HER2-ECD were submitted, and structure prediction was performed using default parameters. Structural models were visualized using PyMOL and Chimera. The A9B5-HER2 interface was analyzed using the PISA server (www.ebi.ac.uk/pdbe/pisa).

### 2.10 Statistics and reproducibility

All graphs and statistical analyses were performed using Excel or GraphPad Prism 10.2.0. Specific tests are described in the relevant method (see above) or figure legends. Quantitative data were analyzed using one-way ANOVA or 2-way ANOVA, as appropriate. Significance thresholds were defined as follows: p < 0.0332 (*), p < 0.0021 (**), p < 0.0002 (***), and p < 0.0001 (****).

## 3 Results

### 3.1 Identification of bpAbs with potent antitumor activity in HER2-positive tumor cells

**Figure 1:**
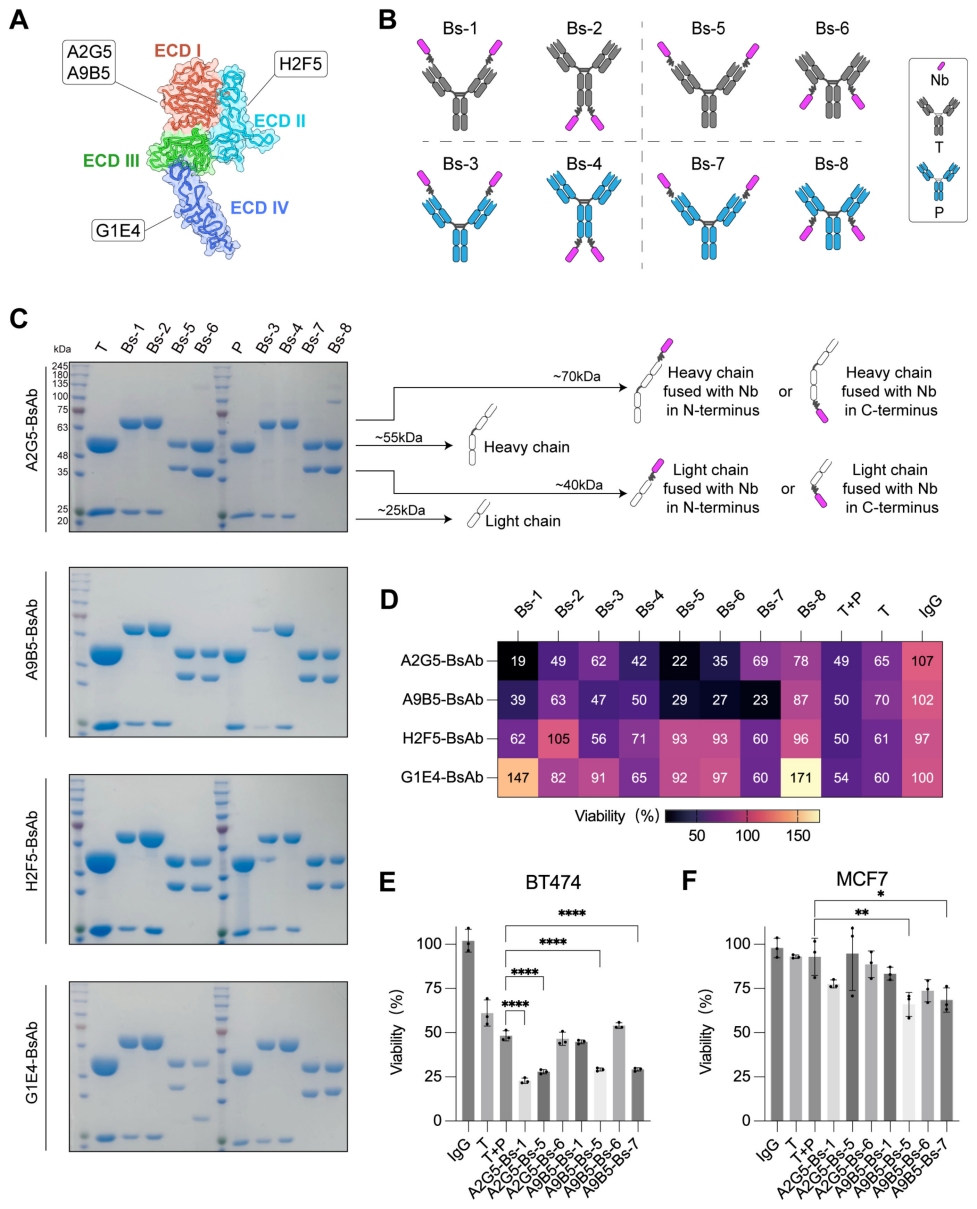
Screening of IgG-VHH bpAbs identifies A9B5-Bs-5 and A9B5-Bs-7 as lead candidates with potent activity in NCI-N87 cells. **(A)** Binding epitopes of high-affinity anti-HER2 nanobodies mapped onto the crystal structure of HER2-ECD monomer (PDB ID: 1N8Z). A2G5 and A9B5 recognized ECD I; H2F5 targeted ECD II; G1E4 recognized ECD IV. Functional characterization of these nanobodies was described previously (49). **(B)** Schematic representation of eight bpAb formats. Nanobodies were fused to different positions of trastuzumab (T) or pertuzumab (P) by (GGGGS)_3_ linker: the N-terminus (Bs-1, Bs-3) or C-terminus (Bs-2, Bs-4) of the heavy chain; the N-terminus (Bs-5, Bs-7) or C-terminus (Bs-6, Bs-8) of the light chain. **(C)** Coomassie-stained SDS-PAGE gel showing high purity of purified biparatopic antibodies. **(D)** The inhibitory effect of bpAbs in NCI-N87 cells (mean of n = 3). **(E, F)** Growth inhibition data in BT474 **(E)** and MCF7 **(F)** cell lines treated with bpAbs (n = 3, mean ± SD). Statistical significance was determined by one-way ANOVA: p < 0.0332 (*), p < 0.0021 (**), p < 0.0002 (***), and p < 0.0001 (****). The p-values were provided in **Supplementary Table S1**. Source data are available in the Source Data file.

BpAbs that bind non-overlapping epitopes on the same molecular target have been reported to exhibit unique functionalities and superior antitumor activity compared with combinations of conventional monospecific antibodies (30, 34, 54–57). Among various bpAbs formats, IgG-VHH fusion - where small (~15 kDa), monomeric VHH domains are fused to IgG scaffolds - represents a highly attractive way for generating stable and functional bpAbs (58–61). To generate novel HER2-targeting bpAbs with favorable developability and manufacturability, high-affinity anti-HER2 nanobodies were employed as antigen-binding partners in constructing IgG-VHH bpAbs. Four nanobodies targeting distinct epitopes of HER2-ECD were previously identified: A2G5 and A9B5 target ECD I, H2F5 targets ECD II, and G1E4 targets ECD IV (**Figure 1A**). These nanobodies demonstrated synergistic inhibitory activity in HER2-expressing tumor cells with acquired resistance (49). Here, we designed eight IgG-VHH bpAbs formats by fusing anti-HER2 nanobodies to the IgG scaffold of trastuzumab or pertuzumab (**Figure 1B**). Nanobodies were fused to different positions of trastuzumab or pertuzumab: the N-terminus (Bs-1, Bs-3) or C-terminus (Bs-2, Bs-4) of the heavy chain; the N-terminus (Bs-5, Bs-7) or C-terminus (Bs-6, Bs-8) of the light chain. A total of 32 IgG-VHH bpAbs were constructed, expressed in Expi293 cells, and purified using standard protocols. All bpAbs exhibited high purity, indicating favorable manufacturability (**Figure 1C**).

BpAbs have been reported to exhibit enhanced antigen affinity and potent inhibitory effect by engaging two epitopes (44, 45). To assess whether these tetravalent IgG-VHH bpAbs benefit from this design, growth inhibition was evaluated in NCI-N87 cells, which express a high level of HER2. BpAbs incorporating ECD I-binding nanobodies (A2G5 and A9B5) demonstrated stronger inhibitory activity than the combination of trastuzumab and pertuzumab (T + P) (**Figure 1D**). In contrast, bpAbs containing ECD II-binding nanobody H2F5 exhibited weaker effects, while those incorporating ECD IV-binding nanobody G1E4 showed agonistic behavior, suggesting that certain configurations may activate HER2 signaling (**Figure 1D**). The seven bpAbs with the strongest growth-inhibitory effects in NCI-N87 cells were selected for subsequent functional screening.

BT474 cells (high HER2 expression) and MCF7 (trastuzumab-resistant) cells were used to identify the most effective bpAbs. In BT474 proliferation assays, A2G5-Bs-1, A2G5-Bs-5, A9B5-Bs-5, and A9B5-Bs-7 demonstrated higher inhibitory activity than T + P (**Figure 1E** and **Supplementary Table S1**). In MCF7 proliferation assays, trastuzumab and T + P exhibited minimal effect on tumor growth, whereas A9B5-Bs-5 and A9B5-Bs-7 mediated significantly greater inhibition compared with T + P (**Figure 1F** and **Supplementary Table S1**). Taken together, A9B5-Bs-5 (bpAb fusing ECD I-binding nanobody to the N-termini of the trastuzumab light chain) and A9B5-Bs-7 (bpAb fusing ECD I-binding nanobody to the N-termini of the pertuzumab light chain) demonstrated significantly greater antitumor effects than T + P across all three cell lines. Therefore, A9B5-Bs-5 and A9B5-Bs-7 were selected for further experiments.

### 3.2 BpAbs bind HER2 with high saturation via recognizing non-overlapping epitopes

**Figure 2:**
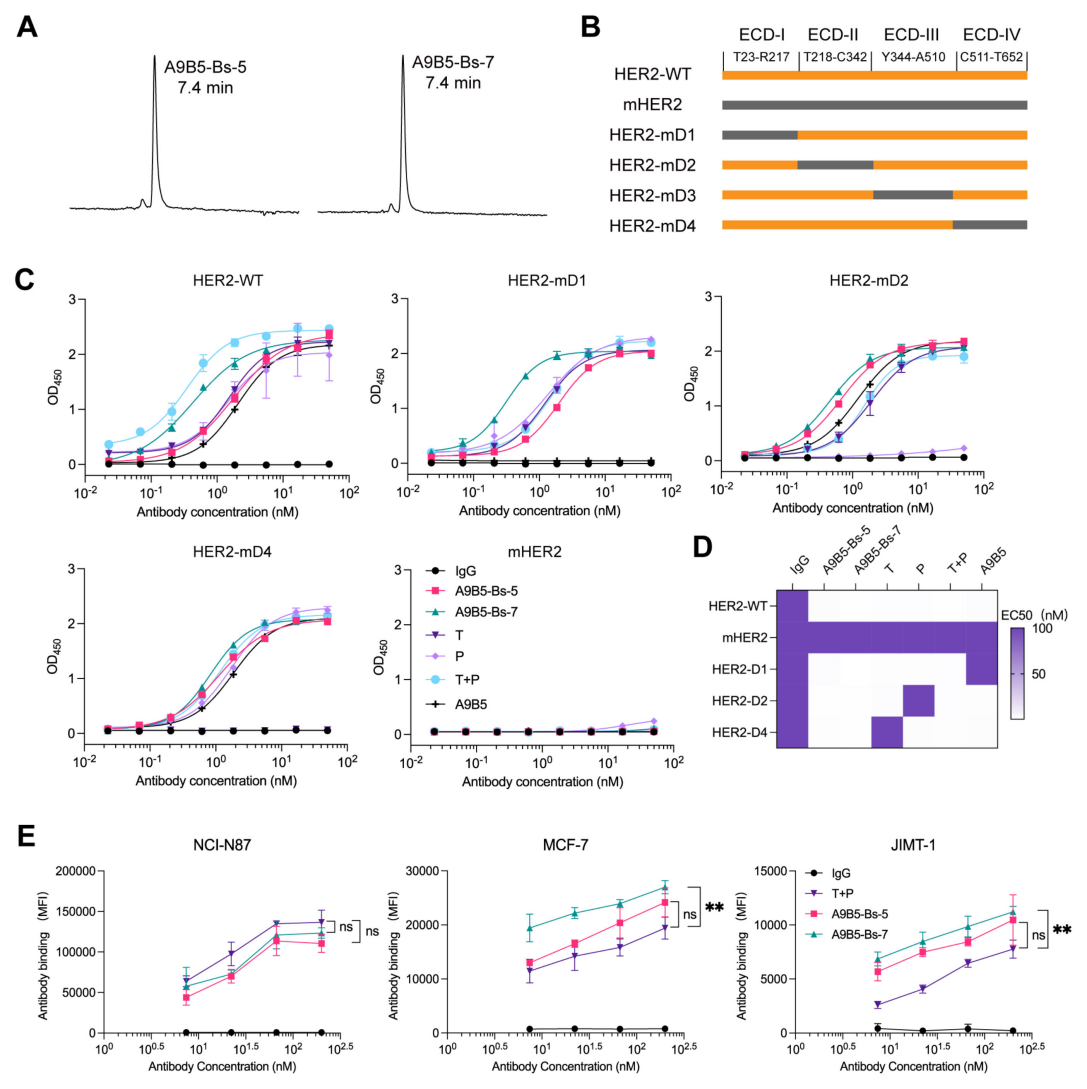
A9B5-Bs-5 and A9B5-Bs-7 bind HER2 with high antibody saturation via recognition of non-overlapping epitopes. **(A)** HPSEC profiles of bpAbs indicating molecular homogeneity. The retention time (RT) was labeled. **(B)** Constructions of chimeric HER2-ECD proteins. Human HER2 domains—ECD I (T23–R217), ECD II (T218–C342), ECD III (Y344–A510), and ECD IV (C511–T652)—were individually replaced with their murine homologs. **(C)** Binding activity of bpAbs against chimeric HER2-ECD proteins. **(D)** Epitope mapping of bpAbs based on the EC_50_ values from ELISA analysis. The EC_50_ values (nM) were shown as a heat map. **(E)** Antibody binding saturation across different HER2-positive tumor cells, measured by FACS. Data represent mean ± SD (n = 3). Statistical significance was determined using one-way ANOVA: p < 0.0332 (*), p < 0.0021 (**), p < 0.0002 (***), and p < 0.0001 (****). The p-values were provided in **Supplementary Table S2**. Source data are available in the Source Data file.

To define how A9B5-Bs-5 and A9B5-Bs-7 engage HER2 molecules, we assessed their homogeneity, epitope specificity, and cell-surface binding capacity. Analytical HPSEC showed that both bpAbs eluted as single major peaks with retention times (RT) of 7.4 min, confirming molecular homogeneity and minimal aggregation (**Figure 2A**). To determine whether the bpAbs could bind two non-overlapping epitopes, we generated a panel of chimeric HER2-ECD proteins in which each human domain (I–IV) was individually replaced by its murine counterpart, as described previously (49) (**Figure 2B**). Because HER2-mD3 expression was undetectable, only HER2-mD1, HER2-mD2, and HER2-mD4 were included in ELISA analysis. Both A9B5-Bs-5 and A9B5-Bs-7 retained specificity to human HER2 and showed negligible binding to murine HER2 (**Figure 2C**). The binding of either bpAb was largely maintained across the domain−swap panel, whereas trastuzumab lost binding to HER2−mD4 and pertuzumab lost binding to HER2−mD2 (**Figure 2C**). ELISA-derived EC_50_ shifts indicated that A9B5-Bs-5 recognizes the epitopes spanning ECD I and ECD IV, whereas A9B5-Bs-7 recognizes ECD I and ECD II (**Figure 2D**).

BpAbs such as zanidatamab can achieve higher cell-surface binding saturation than canonical monospecific antibodies owing to multivalent engagement (30). We next used flow cytometry to assess cell-surface binding capacity across HER2-positive tumor models, including NCI-N87 (high HER2 expression), MCF7 (trastuzumab-resistant), and JIMT-1 (trastuzumab-resistant) cells. In NCI-N87 assays, A9B5-Bs-5 and A9B5-Bs-7 achieved antibody saturation comparable to T + P (**Figure 2E** and **Supplementary Table S2**). In trastuzumab-resistant MCF7 and JIMT-1 cells, A9B5-Bs-7 showed 1.4-fold higher maximal binding than T + P, whereas A9B5-Bs-5 demonstrated comparable antibody saturation to T + P (**Figure 2E** and **Supplementary Table S2**). These results support a model in which A9B5-Bs-5 and A9B5-Bs-7 co-engage non-overlapping HER2 epitopes and achieve superior saturation, likely enabling enhanced receptor internalization and antitumor activity.

### 3.3 BpAbs promote rapid internalization of HER2 receptors

**Figure 3:**
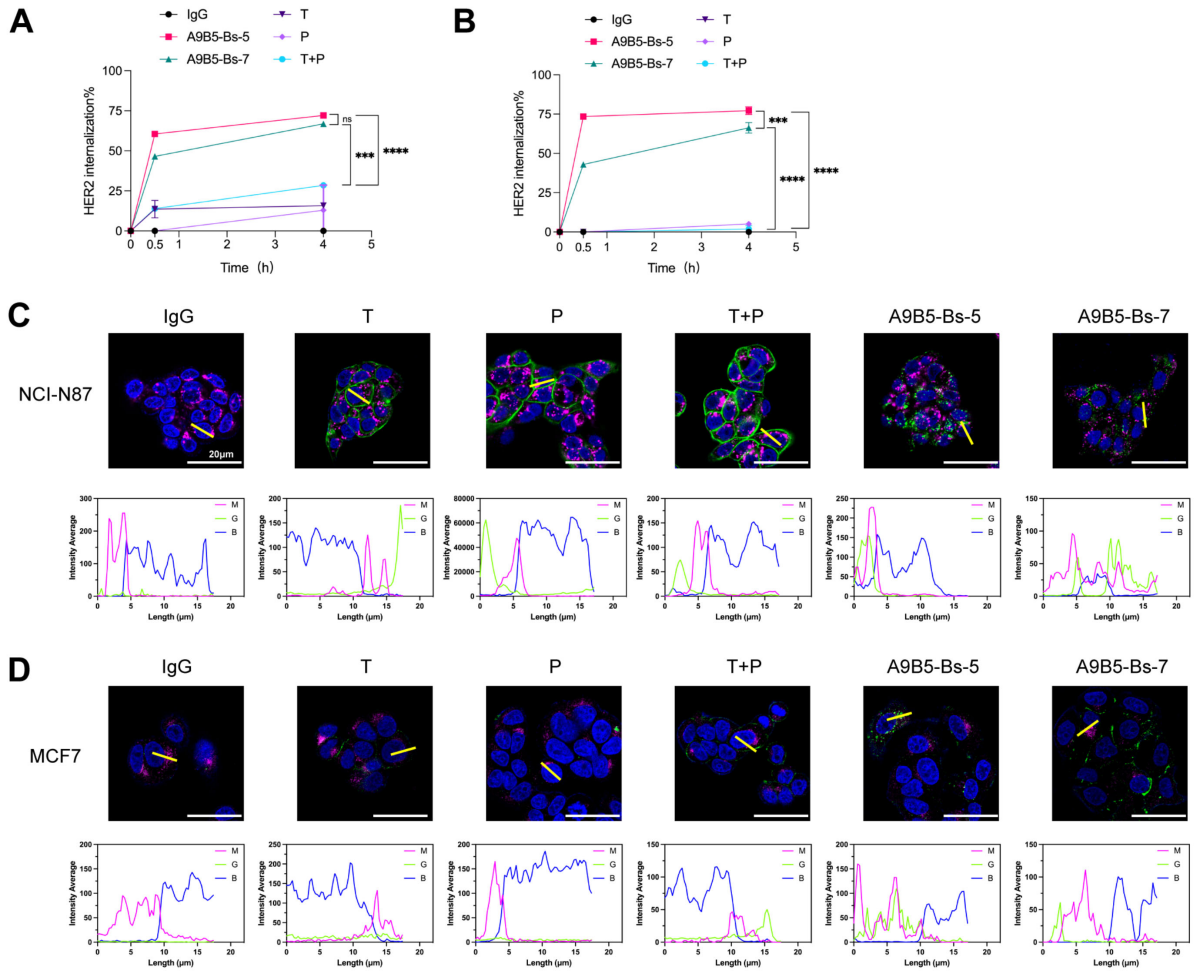
A9B5-Bs-5 and A9B5-Bs-7 facilitate rapid HER2 internalization. **(A, B)** HER2 internalization in NCI-N87 **(A)** and BT474 **(B)** cells following treatment with bpAbs, monospecific antibodies (trastuzumab or pertuzumab), or the trastuzumab + pertuzumab combination (T + P). Cells were incubated with antibodies for 0.5 or 4 h. Internalization was quantified by flow cytometry and displayed as mean percentage ± SD (n=3). Statistical significance was determined using one-way ANOVA: p < 0.0332 (*), p < 0.0021 (**), p < 0.0002 (***), and p < 0.0001 (****). The p-values were provided in **Supplementary Table S3**. **(C, D)** Confocal microscopy studies of antibody-HER2 complexes in NCI-N87 **(C)** or MCF7 **(D)** cells at 4 h post-treatment. Antibody-HER2 complexes were visualized using anti-human Fc staining (green), lysosomes by LAMP1 (magenta), and the nucleus by DAPI (blue). Scale bars, 20 μm. Fluorescence intensity from magenta (M), green **(G)**, and blue **(B)** channels was quantified using ImageJ to assess colocalization of internalized complexes with lysosomes. Source data are available in the Source Data file.

Receptor clustering at the cell surface induced by bpAbs has been reported to accelerate rapid receptor internalization, suppress recycling, and promote lysosomal degradation (30, 45, 62). To test whether A9B5-Bs-5 and A9B5-Bs-7 enhance HER2 internalization, we treated BT474 and NCI-N87 cells with anti-HER2 antibodies and quantified cell-surface HER2 by FACS over 0.5–4 h. In NCI-N87 cells, trastuzumab, pertuzumab, and their combination (T + P) showed limited internalization (< 30%), whereas both bpAbs drove rapid and substantially greater HER2 loss from the surface (**Figure 3A** and **Supplementary Table S3**). In BT474 cells, trastuzumab, pertuzumab, and T+P induced little to no internalization, whereas both bpAbs again triggered markedly greater HER2 loss (**Figure 3B** and **Supplementary Table S3**). A9B5-Bs-5 mediated more pronounced internalization than A9B5-Bs-7.

We next used confocal microscopy to visualize the intracellular trafficking in two cell lines. In NCI-N87 cells, single antibodies (trastuzumab or pertuzumab) remained evenly distributed at the cell membrane, and T + P formed some surface clusters and regions of continuous membrane staining (**Figure 3C**). In contrast, A9B5-Bs-5 and A9B5-Bs-7 formed large intracellular complexes that co-localized with lysosomes (**Figure 3C**). In MCF7 cells (low HER2 expression), trastuzumab, pertuzumab, or their combination showed weak surface-binding with minimal internalization (**Figure 3D**). A9B5-Bs-7 yielded enhanced surface-binding with moderate internalization, whereas A9B5-Bs-5 showed minimal surface staining yet pronounced internalization with strong co-stain to the lysosomal marker LAMP1. Taken together, these data demonstrated that both bpAbs induce faster and more extensive HER2 internalization than trastuzumab, pertuzumab, and their combination, and that the magnitude of bpAb−driven internalization depends on HER2 expression level.

### 3.4 BpAbs suppress the growth of trastuzumab-resistant tumor cells

**Figure 4:**
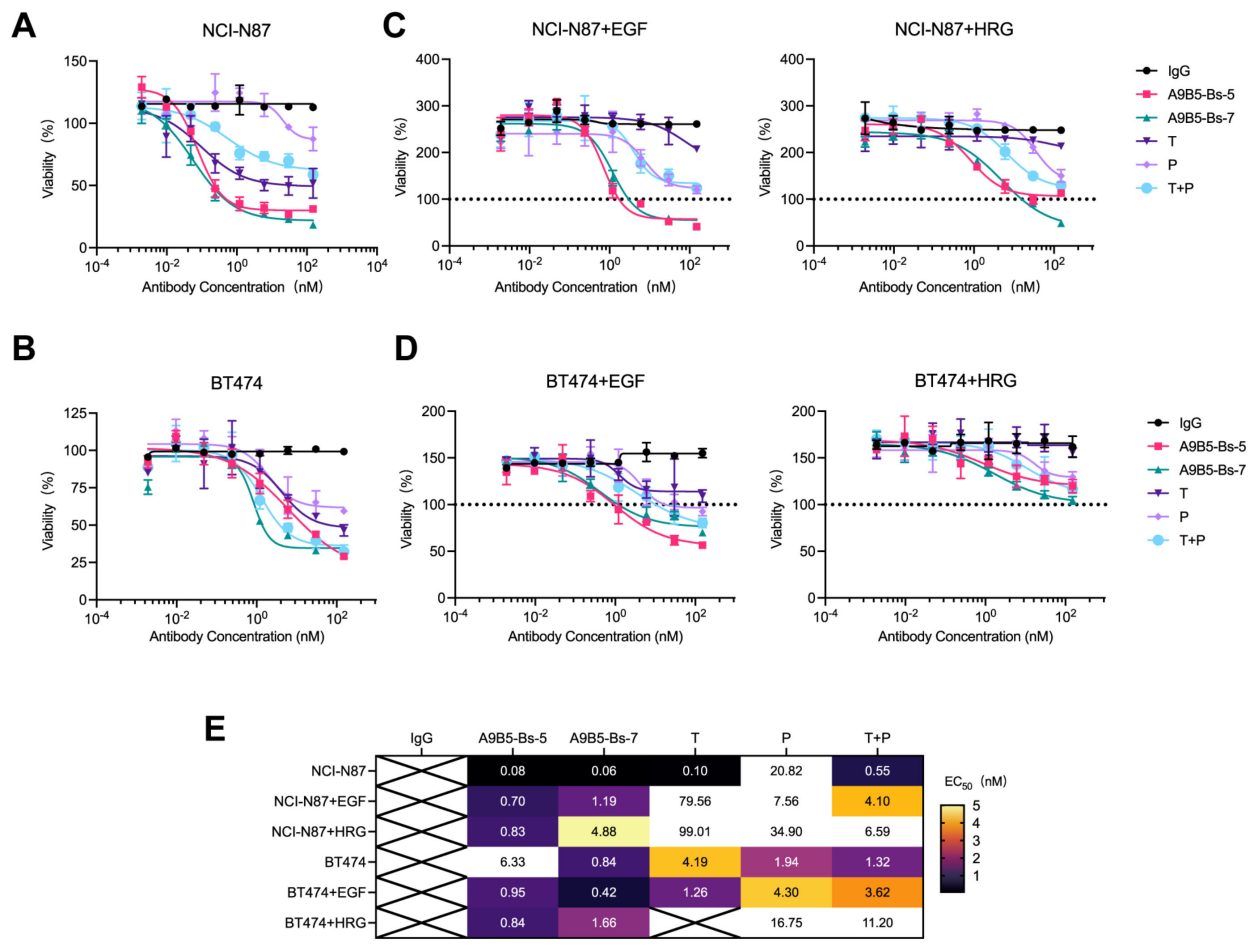
A9B5-Bs-5 and A9B5-Bs-7 inhibit the growth of trastuzumab-resistant HER2-positive cancer cells. **(A, B)** Growth inhibition in NCI-N87 **(A)** and BT474 cells **(B)** treated with bpAbs in the absence of ligands, measured by CCK-8 assay. **(C, D)** Growth inhibition in NCI-N87 **(C)** and BT474 cells **(D)** treated with bpAbs in the presence of EGF or HRG, measured by CCK-8 assay. Serum-starved cells were incubated with anti-HER2 diluted antibodies in the presence of 1 nM HRG or 5 nM EGF. Horizontal dotted line (black) represents viability of non-treated cells referenced to 100%. Data represent mean ± SD (n = 3). **(E)** Heat map of EC_50_ values (nM) derived from CCK-8 assays showing dose-dependent inhibition by bpAbs. Source data are available in the Source Data file.

We next assessed whether the high binding saturation and HER2 internalization conferred by bpAbs translate into anti-proliferative effects in HER2-expressing cell lines, particularly trastuzumab-resistant tumor cells. We first examined ligand-independent antitumor activity by evaluating tumor cell viability after anti-HER2 antibody treatment. Both bpAbs caused concentration-dependent reduction in viability in NCI-N87 and BT474 cells (**Figures 4A, B**). A9B5-Bs-7 was more potent (EC_50_ < 1 nM) than trastuzumab, pertuzumab, and their combination (T + P) in two cell lines (**Figure 4E**). A9B5-Bs-5 displayed comparable or superior activity to T + P or single antibodies (**Figure 4E**). Notably, A9B5-Bs-5 exerted significantly stronger inhibition (EC_50_ < 1 nM) than trastuzumab, pertuzumab, and T + P in NCI-N87 cells, whereas in BT474 cells its effect was weaker than T + P but comparable with either single antibody (**Figures 4B, E**).

We next evaluated antitumor activity under epidermal growth factor (EGF)- and heregulin (HRG)-driven growth conditions. Ligand-driven activation of HER2-EGFR and HER2-HER3 heterodimers is known to contribute to trastuzumab resistance (13, 63). As expected, the ECD IV-targeting antibody trastuzumab exhibited limited efficacy under these conditions (**Figures 4C, D**). The ECD II-targeting antibody pertuzumab, which disrupts HER2 heterodimerizations, showed stronger inhibition than trastuzumab in ligand-stimulated cells. Consistent with previous findings, T + P provided greater growth suppression than either single antibody. Both bpAbs, A9B5-Bs-5 and A9B5-Bs-7, achieved markedly stronger ligand-driven inhibition than trastuzumab, pertuzumab, and T + P (**Figures 4C, D**). A9B5-Bs-5 was particularly active, with EC_50_ values below 1 nM under both EGF and HRG stimulation in both cell lines (**Figure 4E**). In summary, the bpAbs mediate potent inhibition of ligand-independent and ligand-driven growth in HER2-expressing cell lines. In ligand−stimulated, trastuzumab−resistant cells, their efficacy exceeded that of T + P (**Figure 4E**).

### 3.5 Structural modeling supports a *trans*-binding mode and HER2 clustering mechanism

**Figure 5:**
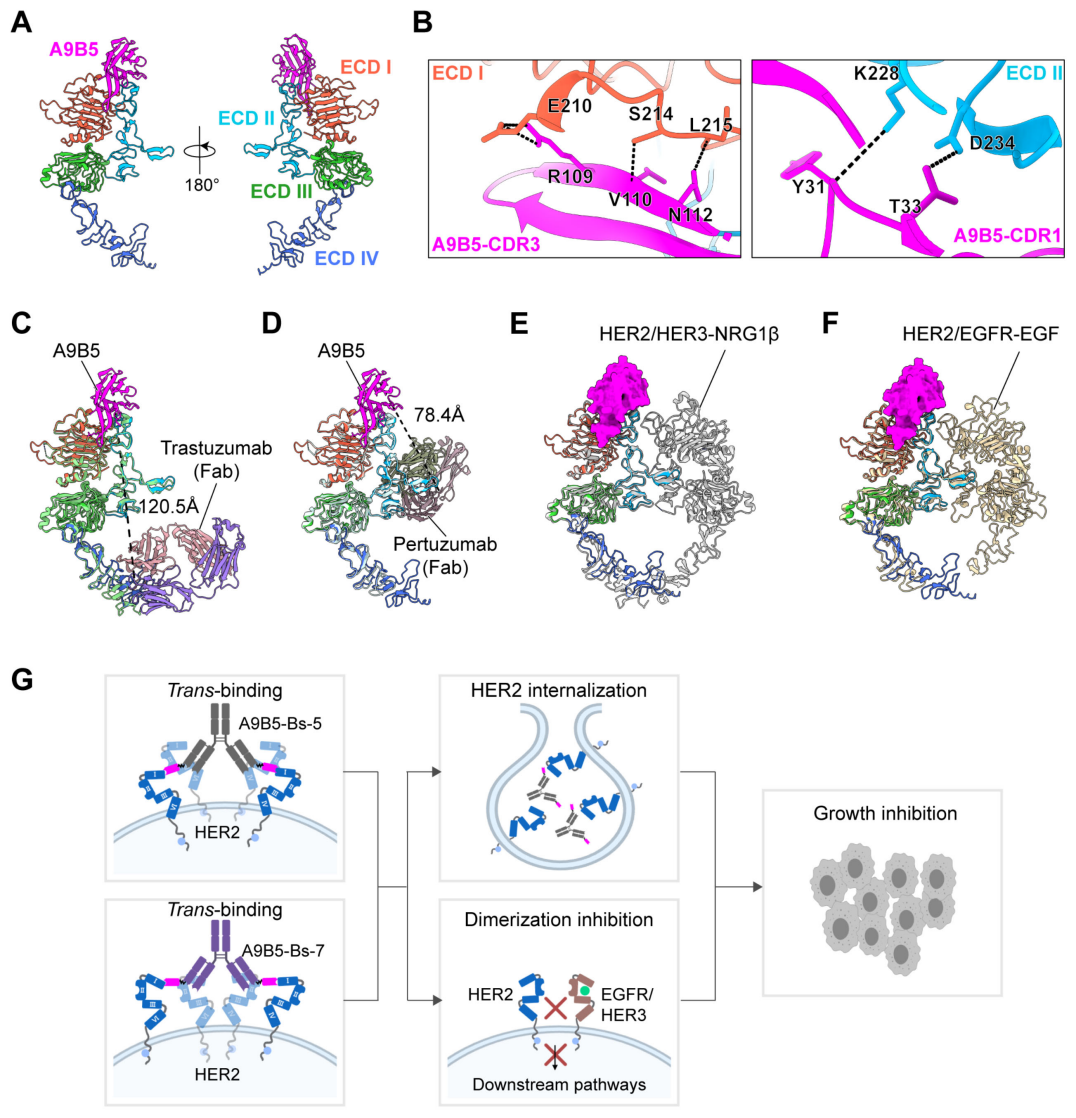
Structural modeling of HER2-ECD complexed with bpAbs supports a trans-binding mechanism. **(A)** Predicted structure of A9B5-HER2 complex generated by Alphafold 3. Nanobody A9B5 was shown in magenta. The ECD I-IV of HER2 were colored in tomato, deep sky blue, lime green, and royal blue, respectively. The predicted structure is available in the Source Data file. **(B)** Interface analysis of the A9B5-HER2 complex. Potential hydrogen bonds (< 4 Å) were indicated by black dashed lines. The detailed interaction sites were provided in **Supplementary Table S4**. **(C)** Structural alignment of the A9B5-HER2 complex with the trastuzumab-HER2 complex (PDB ID: 1N8Z). Trastuzumab Fab was shown in medium purple (light chain) and light pink (heavy chain). Dashed black lines indicate the distance between the C-terminus of A9B5 and N-terminus of trastuzumab light chain. **(D)** Structural alignment of the A9B5-HER2 complex with the pertuzumab-HER2 complex (PDB ID: 1S78). Dashed black lines indicate the distance between the C-terminus of A9B5 and the N-terminus of the pertuzumab light chain. **(E)** Superposition of the A9B5-HER2 complex and the HER2-HER3-NRG1β dimer complex (PDB ID: 7MN5). The HER2-HER3-NRG1β dimer complex was shown in light gray. **(F)** Superposition of the A9B5-HER2 complex and the HER2-EGFR-EGF dimer complex (PDB ID: 8HGO). The HER2-EGFR-EGF dimer complex was shown in wheat. **(G)** Schematic model illustrating the proposed *trans*-binding mechanism. A9B5-Bs-5 and A9B5-Bs-7 may promote HER2 clustering by engaging HER2 molecules on adjacent receptors, thereby facilitating receptor internalization and suppressing HER2-driven heterodimerization. The image was created with *MedPeer.cn*.

To define how the engineered biparatopic formats engage HER2, we modeled the nanobody A9B5 in complex with HER2-ECD using AlphaFold 3. The top−ranked model positioned A9B5 across a composite surface spanning ECD I and ECD II (**Figure 5A**). Interface residues were analyzed with the PISA server, which suggested six putative hydrogen−bonding contacts. Representative interactions involved three residues (E210, S214, and L215) on ECD I and two residues (K228 and D234) on ECD II (**Figure 5B** and **Supplementary Table S4**). The cross−subdomain footprint indicates that A9B5 recognizes a surface bridging ECD I and ECD II, potentially constraining local interdomain flexibility.

To assess whether an A9B5 moiety covalently linked to trastuzumab could engage the same HER2 molecules in a *cis*-binding pattern, we aligned the A9B5-HER2 complex with the trastuzumab−HER2 complex (PDB ID: 1N8Z) (**Figure 5C**). The modeled C−terminus of A9B5 lay ~120.5 Å from the N−terminus of the trastuzumab light chain. Because A9B5−Bs−5 incorporates a flexible (GGGGS)_3_ linker with an estimated maximal reach of ~57 Å, this separation markedly exceeds the linker span, making simultaneous *cis* co-occupancy of A9B5 and trastuzumab epitopes on a single HER2 molecule unlikely. These constraints, therefore, favor a *trans*-binding mode in which A9B5 binds one molecule and the trastuzumab Fab engages a second. An analogous alignment with the pertuzumab−HER2 structure (PDB ID: 1S78) yielded a ~78.4 Å distance between the C−terminus of A9B5 and the N−terminus of the pertuzumab light chain (**Figure 5D**). This also exceeds the ~57 Å reach of the (GGGGS)_3_ linker in A9B5−Bs−7, again arguing against efficient *cis* co-occupancy and supporting a *tran*-binding mode.

To examine whether the A9B5 epitope remains accessible in ligand-activated receptor states, the A9B5-HER2 model was superposed onto structures of the HER2-HER3-NRG1β dimer (PDB ID: 7MN5) and the HER2-EGFR-EGF dimer (PDB ID: 8HGO) (**Figures 5E, F**). The A9B5 footprint remained largely solvent-exposed in both heterodimer conformations, with minimal occlusion by the partnering receptor ectodomains. Approach angles may be modestly restricted near the DA of ECD II; however, most of the interface appears accessible, suggesting that A9B5−containing bpAbs could bind HER2 even within ligand−driven heterodimers.

Together, these structural analyses support a *trans*−binding mechanism (**Figure 5G**). The (GGGGS)_3_ linker within a single antibody arm is insufficient to span the >57 Å separations required for *cis*-engagement of A9B5 with either trastuzumab or pertuzumab epitopes on the single HER2 molecule. Instead, each arm of A9B5−Bs−5 or A9B5−Bs−7 is predicted to bridge two HER2 molecules. Because the full−length IgG format is bivalent, a single bpAb could engage up to four HER2 molecules (two per arm), promoting local receptor clusters on the cell surface. Such clustering would be expected to induce receptor internalization and degradation and to sterically interfere with HER2−mediated heterodimerization, providing a structural rationale for the enhanced antitumor activity observed for these bpAbs in trastuzumab−resistant cells (**Figure 4E**).

## 4 Discussion

HER2-targeted therapies have revolutionized the therapeutic landscape for breast, gastric, and other solid tumors. However, treatment resistance remains a major clinical obstacle (5, 33, 64–71). To overcome the limitations of current standard-of-care therapies, we engineered two bpAbs, A9B5−Bs−5 and A9B5−Bs−7, tetravalent IgG-VHH constructs that bind non-overlapping epitopes on the HER2-ECD. These bpAbs exhibited enhanced functional properties compared to the clinically approved anti-HER2 antibodies trastuzumab and pertuzumab, either alone or in combination (T + P). A9B5−Bs−5 and A9B5−Bs−7 bound to cell-surface HER2 with high saturation and induced rapid HER2 internalization—an effect not observed with trastuzumab, pertuzumab, or T + P. Furthermore, both bpAbs exhibited superior or comparable growth-inhibition relative to T + P in both ligand-independent and ligand-driven tumor models. Structural modeling suggests that these bpAbs engage HER2 in a *trans*-binding pattern, promoting the formation of large receptor clusters.

The development of novel bsAbs is guided by a deep understanding of biological mechanism, which must be aligned with optimal formats, affinity profiles and epitope selection (50). IgG-VHH fusions, which combine IgG scaffolds with small nanobodies (VHHs), are highly attractive due to their structural simplicity, high stability, and favorable biophysical properties (60, 61, 72, 73). VHH domains are inherently monomeric and do not require pairing with a light chain, minimizing the risk of mispairing or aggregation that often compromises the performance of scFv-IgG formats. Leveraging these design principles, we constructed symmetric IgG-VHH fusions by fusing HER2-specific nanobodies to the IgG scaffolds of trastuzumab and pertuzumab (**Figure 1B**). A comprehensive comparison of different IgG-VHH bpAb architectures identified A9B5-Bs-5 and A9B5-Bs-7 as lead candidates, both exhibiting high purity, low aggregation propensity, and favorable manufacturability following Protein A purification (**Figures 1C** and **2A**).

While previous studies have suggested that VHH fusion to the heavy chain is generally superior to light-chain fusion, our constructs defy this convention (58, 60). A9B5-Bs-5 and A9B5-Bs-7, in which the nanobody A9B5 is fused to the N-terminus of light chain via a (GGGGS)_3_ flexible linker, retained high binding affinity (EC_50_ < 2 nM) for both wild-type and chimeric HER2-ECD proteins and achieved high binding saturation on HER2-positive tumor cells (**Figures 2D, E**). These findings may be attributed to the flexible linker minimizing steric hindrance and the enhanced avidity conferred by dual epitope engagement (74, 75). Interestingly, certain configurations (e.g., G1E4-Bs-1 and G1E4-Bs-8) exhibited agonist activity (**Figure 1D**), consistent with observations from DVD-Ig formats that can aberrantly activate HER2 signaling depending on the spatial orientation of their binding domains (76). The underlying mechanisms of this agonistic behavior remain to be elucidated.

Resistance to anti-HER2 therapies arises from multiple mechanisms, including HER family alterations, masking of HER2 epitope, and activation of compensatory pathways. These multi-faceted resistance mechanisms significantly limit the effectiveness of traditional HER2-targeted therapies, which predominantly rely on single-modal MOA (34, 55). As a distinct subclass of bispecific antibodies, bpAbs represent a promising strategy for overcoming therapeutic resistance through dual HER2 blockade (34, 55, 71, 77–79). Several bpAbs currently in late-stage clinical development, including zanidatamab and anbenitamab, have shown favorable outcomes in HER2-expressing tumors (27, 31, 80–83). Engagement of non-overlapping epitopes enables multiple MOAs, including enhanced antibody saturation, receptor clustering, and rapid internalization (30). Our A9B5-containing bpAbs leverage these mechanisms and achieved greater or comparable antitumor efficacy than trastuzumab plus pertuzumab. Notably, most anti-HER2 bpAbs in development are derived from trastuzumab and pertuzumab, targeting common epitopes on ECD II and ECD IV (56, 84, 85). Recent structural studies indicate that targeting alternative domains such as ECD I and ECD III may improve blockade of HER2-driven oncogenic signaling (38, 39, 86). The A9B5 nanobody, obtained from a synthetic VHH library, binds ECD I with high affinity and exhibits strong synergy with trastuzumab in resistant tumor models (49). We hypothesize that ECD I engagement may facilitate additional binding modes, improved compatibility, and larger-scale receptor clustering. Structural predictions indicate that the A9B5 epitope remains accessible in ligand-activated HER2 conformations (**Figures 5E, F**), and in ligand-dependent models, A9B5−containing bpAbs demonstrated potent growth inhibition (**Figure 4E**). Whether this inhibitory activity stems from the disruption of HER2-containing heterodimers remains to be investigated. In addition, structural modeling supports a *trans*-binding mode of HER2 engagement by A9B5−containing bpAbs, potentially driving the formation of high-order HER2 clusters and facilitating internalization (**Figure 5G**). Similar *trans*-binding modality is employed by other HER2-targeting bpAbs, such as zanidatamab, which recognizes ECD II and ECD IV and promotes receptor reorganization to enhance CDC (30). Whether A9B5−based constructs can similarly recruit Fc-mediated effector functions remains to be determined. Moreover, although domain-swap ELISA primarily indicated binding to ECD I, this reflects the fact that key ECD II interface residues predicted by modeling (K228 and D234) are conserved between human and murine HER2. Thus, the experimental results mainly highlight species-specific determinants, whereas structural modeling provides a broader view of the composite epitope across ECD I–II. Taken together, these complementary approaches suggest that A9B5 engages a cross-subdomain surface that may stabilize distinct HER2 conformations and facilitate receptor clustering.

This study has several limitations. First, the predicted nanobody-HER2 interactions were derived from AlphaFold 3 structural models. Although AlphaFold provides near-experimental accuracy, definitive structural insights will require confirmation via cryo-electron microscopy or X-ray crystallography (53, 87). Second, the antitumor efficacy and MOAs of bpAbs need to be further verified in more cell lines. Third, the antitumor efficacy of A9B5-based bpAbs needs to be further validated in *in vivo* models to support their translational potential.

## 5 Conclusion

In conclusion, we engineered two IgG-VHH bpAbs, A9B5-Bs-5 and A9B5-Bs-7. In *in vitro* models, these antibodies demonstrated superior HER2 binding, receptor internalization, and growth inhibition compared to trastuzumab, pertuzumab, or their combination. Our findings highlight ECD I engagement as a viable design strategy for next-generation biparatopic anti-HER2 antibodies, potentially enabling functional activities not achieved by conventional monospecific agents. The ability of these antibodies to retain activity in trastuzumab-resistant cell models supports their potential utility in overcoming resistance mechanisms, and warrants further investigation in preclinical *in vivo* studies to assess their therapeutic applicability.

## Data Availability

The datasets presented in this study can be found in online repositories. The names of the repository/repositories and accession number(s) can be found in the article/**Supplementary Material**.
