# Supplementary material for: Engineering HER2-targeted biparatopic antibodies to promote receptor internalization and restore antitumor efficacy
Source: Front Immunol. 2025 Nov 7;16:1711433. doi: 10.3389/fimmu.2025.1711433 (PMC12634555; doi:10.3389/fimmu.2025.1711433)
Supplement: Supplementary file 2 [file Table2.docx]

Supplementary Material

# Supplementary Table 1. P-values for comparisons of percent viability at different treatments in cancer cell lines

| **Comparison^a^** | **BT474** | | **MCF7** | |
| --- | --- | --- | --- | --- |
|  | **Significance** | **P Value** | **Significance** | **P Value** |
| T+P vs. IgG | **** | <0.0001 | ns | 0.9854 |
| T+P vs. T | ** | 0.002 | ns | >0.9999 |
| T+P vs. A2G5-Bs-1 | **** | <0.0001 | ns | 0.245 |
| T+P vs. A2G5-Bs-5 | **** | <0.0001 | ns | >0.9999 |
| T+P vs. A2G5-Bs-6 | ns | 0.9948 | ns | 0.9958 |
| T+P vs. A9B5-Bs-1 | ns | 0.8012 | ns | 0.72 |
| T+P vs. A9B5-Bs-5 | **** | <0.0001 | ** | 0.0098 |
| T+P vs. A9B5-Bs-6 | ns | 0.3 | ns | 0.0931 |
| T+P vs. A9B5-Bs-7 | **** | <0.0001 | * | 0.0207 |

^a^ One-way ANOVA was used to calculate p-values for comparisons. Statistical significance was defined as follows: p < 0.0332 (*), p < 0.0021 (**), p < 0.0002 (***), and p < 0.0001 (****).

# Supplementary Table 2. P-values for comparison of antibody binding (MFI) between biparatopic antibodies (200nM) and T + P (200 nM + 200 nM) across different cell lines

| **Comparison^a^** | **NCI-N87** | | **MCF-7** | | **JIMT-1** | |
| --- | --- | --- | --- | --- | --- | --- |
|  | **Significance** | **P Value** | **Significance** | **P Value** | **Significance** | **P Value** |
| T+P vs. A9B5-Bs-5 | ns | 0.0525 | ns | 0.0522 | ns | 0.1076 |
| T+P vs. A9B5-Bs-7 | ns | 0.331 | ** | 0.0073 | * | 0.0473 |

^a^ One-way ANOVA was performed to calculate p-values for comparisons of antibody binding, measured by median fluorescence intensity (MFI), in NCI-N87, MCF-7, and JIMT-1 cells following antibody treatment. Statistical significance was defined as follows: p < 0.0332 (*), p < 0.0021 (**), p < 0.0002 (***), and p < 0.0001 (****).

# Supplementary Table 3. P-values for comparisons of receptor internalization in cancer cell lines

| **Comparison^a^** | **NCI-N87** | | **BT474** | |
| --- | --- | --- | --- | --- |
|  | **Significance** | **P Value** | **Significance** | **P Value** |
| A9B5-BS-5 vs. A9B5-BS-7 | ns | 0.9079 | *** | 0.0008 |
| A9B5-BS-5 vs. T | **** | <0.0001 | **** | <0.0001 |
| A9B5-BS-5 vs. P | **** | <0.0001 | **** | <0.0001 |
| A9B5-BS-5 vs. T+P | **** | <0.0001 | **** | <0.0001 |
| A9B5-BS-7 vs. T | **** | <0.0001 | **** | <0.0001 |
| A9B5-BS-7 vs. P | **** | <0.0001 | **** | <0.0001 |
| A9B5-BS-7 vs. T+P | *** | 0.0001 | **** | <0.0001 |

^a^ One-way ANOVA was used to calculate p-values for comparisons of HER2 internalization following antibody treatment in NCI-N87 and BT474 cells. Statistical significance was defined as follows: p < 0.0332 (*), p < 0.0021 (**), p < 0.0002 (***), and p < 0.0001 (****).

# Supplementary Table 4. Hydrogen bonding contacts between the CDR regions of A9B5 and HER2-ECD

| **Epitope on HER2-ECD** | **A9B5 residue** | **Distance (Å)** |
| --- | --- | --- |
| LYS 228[ NZ ] ^a^ | TYR 31[ O ] | 3.83 |
| SER 214[ OG ] | VAL 110[ O ] | 2.60 |
| GLU 210[ OE2] | ARG 109[ NH1] | 3.52 |
| GLU 210[ OE2] | ARG 109[ NH2] | 3.50 |
| LEU 215[ O ] | ASN 112[ ND2] | 2.34 |
| ASP 234[ OD2] | THR 33[ OG1] | 2.23 |

^a^ The atoms involved in the hydrogen bonding contact were speciﬁed in parentheses.
