# Supplementary figures and images for: Engineering HER2-targeted biparatopic antibodies to promote receptor internalization and restore antitumor efficacy
Source: Front Immunol. 2025 Nov 7;16:1711433. doi: 10.3389/fimmu.2025.1711433 (PMC12634555; doi:10.3389/fimmu.2025.1711433)

**Figure.1C**

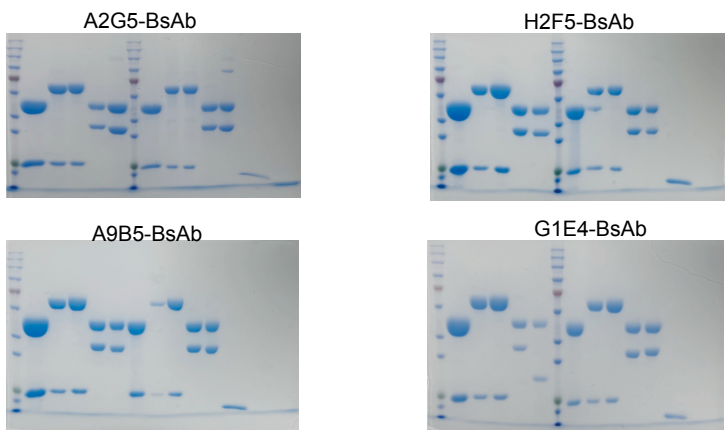

**Figure.3C**

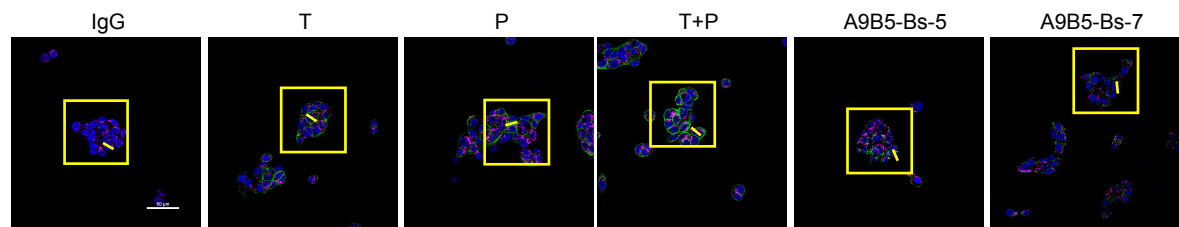

**Figure.3D**

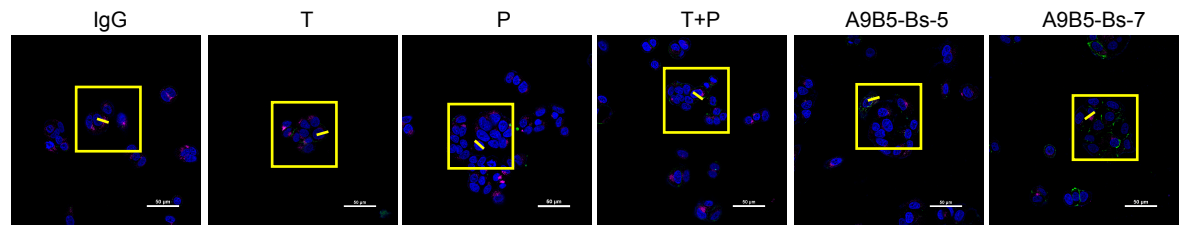

Supplement: Supplementary file 3 [file DataSheet1.pdf]
